# Degenerative Cervical Myelopathy: Development and Natural History [AO Spine RECODE-DCM Research Priority Number 2]

**DOI:** 10.1177/21925682211036071

**Published:** 2022-02-17

**Authors:** Aria Nouri, Enrico Tessitore, Granit Molliqaj, Torstein Meling, Karl Schaller, Hiroaki Nakashima, Yasutsugu Yukawa, Josef Bednarik, Allan R. Martin, Peter Vajkoczy, Joseph S. Cheng, Brian K. Kwon, Shekar N. Kurpad, Michael G. Fehlings, James S. Harrop, Bizhan Aarabi, Vafa Rahimi-Movaghar, James D. Guest, Benjamin M. Davies, Mark R. N. Kotter, Jefferson R. Wilson

**Affiliations:** 1 Division of Neurosurgery, Geneva University Hospitals, University of Geneva, Geneva, Switzerland; 2 Department of Orthopedic Surgery, Nagoya University Graduate School of Medicine, Nagoya, Japan; 3 Department of Orthopedic Surgery, Wakayama Medical University, Wakayama, Japan; 4 Department of Neurology, University Hospital Brno and Faculty of Medicine, Masaryk University, Brno, Czech Republic; 5 Department of Neurosurgery, University of California Davis, Sacramento, CA, USA; 6 Department of Neurosurgery, Charité Universitätsmedizin, Berlin, Germany; 7 Department of Neurosurgery, University of Cincinnati, Cincinnati, OH, USA; 8 Vancouver Spine Surgery Institute, Department of Orthopedics, The University of British Columbia, Vancouver, British Columbia, Canada; 9 Department of Neurosurgery, Medical College of Wisconsin, Wauwatosa, WI, USA; 10 Division of Neurosurgery and Spine Program, University of Toronto, Ontario, Canada; 11 Department of Neurological Surgery, Thomas Jefferson University, Philadelphia, PA, USA; 12 Department of Neurosurgery, University of Maryland, Baltimore, MD, USA; 13 Department of Neurosurgery, Sina Trauma and Surgery Research Center, Tehran University of Medical Sciences, Tehran, Iran; 14 Department of Neurosurgery and The Miami Project to Cure Paralysis, The Miller School of Medicine, University of Miami, FL, USA; 15 Department of Neurosciences, University of Cambridge, Cambridge, United Kingdom; 16 Myelopathy.org, International Charity for Degenerative Cervical Myelopathy, United Kingdom

**Keywords:** cervical spondylotic myelopathy (CSM), cord compression, ossification of the posterior longitudinal ligament (OPLL), progression, risk factors

## Abstract

**Study Design:**

Narrative review.

**Objectives:**

To discuss the current understanding of the natural history of degenerative cervical myelopathy (DCM).

**Methods:**

Literature review summarizing current evidence pertaining to the natural history and risk factors of DCM.

**Results:**

DCM is a common condition in which progressive arthritic disease of the cervical spine leads to spinal cord compression resulting in a constellation of neurological symptoms, in particular upper extremity dysfunction and gait impairment. Anatomical factors including cord-canal mismatch, congenitally fused vertebrae and genetic factors may increase individuals’ risk for DCM development. Non-myelopathic spinal cord compression (NMSCC) is a common phenomenon with a prevalence of 24.2% in the healthy population, and 35.3% among individuals >60 years of age. Clinical radiculopathy and/or electrophysiological signs of cervical cord dysfunction appear to be risk factors for myelopathy development. Radiological progression of incidental Ossification of the Posterior Longitudinal Ligament (OPLL) is estimated at 18.3% over 81-months and development of myelopathy ranges between 0-61.5% (follow-up ranging from 40 to 124 months between studies) among studies. In patients with symptomatic DCM undergoing non-operative treatment, 20-62% will experience neurological deterioration within 3-6 years.

**Conclusion:**

Current estimates surrounding the natural history of DCM, particularly those individuals with mild or minimal impairment, lack precision. Clear predictors of clinical deterioration for those treated with non-operative care are yet to be identified. Future studies are needed on this topic to help improve treatment counseling and clinical prognostication.

## Introduction

Degenerative Cervical Myelopathy (DCM) occurs when progressive arthritic/spondylotic changes narrow the cervical spinal canal, leading to spinal cord compression and progressive spinal cord impairment.^1-3^ The clinical manifestations of this disease exist on a spectrum of severity; while severely affected patients may be unable to walk or use their hands, mildly affected patients may experience only minor symptoms and have a good quality of life. Understanding of the rate at which patients move along this continuum without operative treatment—the so-called natural history of DCM—remains limited.

Knowledge regarding prognosis for progression is vital in the context of DCM since the goal of operative intervention is to arrest symptomatic progression and functional decline. In evaluating whether to perform surgery on a DCM patient with relatively mild symptoms, the risks of cervical spine surgery are justified if that individual is at high-risk for deterioration if managed non-operatively. Conversely, if the risks of deterioration are low, it makes sense to avoid upfront surgery and closely follow the patient. Therefore, our knowledge surrounding natural history and prognosis is essential for DCM-related treatment decision making.

Apart from patients with symptomatic myelopathy, another critical question relates to the prognosis of individuals with cervical spinal cord compression but without myelopathy or with minimal impairment. In such individuals–who are increasingly recognized due to the ubiquity of neuroimaging–it is important to understand the risk of myelopathy development, or potentially catastrophic spinal cord injury, for purposes of patient counseling and treatment planning. The lack of clear clinical guidance in this regard was highlighted in a series of systematic review and practice guidelines on DCM published in 2017.^4^

Here, we provide an overview of topics pertaining to the natural history of DCM to inform prognosis and decision-making. We have summarized the existing evidence and highlighted key knowledge gaps and important opportunities for research. Wherever possible, we focus discussions on more recent and higher quality (prospective) studies. A summary of key natural history studies is highlighted in Table 1.

**Table 1.** Summary Table of Natural History Studies. Adapted and Modified From *Karadimas, Erwin ^87^ and Tetreault, Karadimas.*^*5*^

Authors & study design	Demographics	Follow-up, mean (%)	Inclusion criteria	Outcome measure	Prognostic factors noted (Significant factors)	Natural history estimate
Barnes and Saunders^6^ Retrospective cohort	N = 76 Mean age = 65yr Male = 71%	8.2 yr (59%)	1. Myelopathy with evidence of corticospinal tract dysfunction in the legs with or without sensory involvement or radiculopathy 2. Plain radiological changes of cervical spondylosis 3. Myelographic evidence of a complete or partial block to the flow of contrast medium in the cervical spine 4. No other reasonable diagnosis that had manifested itself on follow-up examination	Change in Nurick grade Better, same or worse Nurick grade	Patients that deteriorated: - Were more often women (*P* = .01) - Range of neck movement is greater (*P* < .05) - Range of head movement is also higher (*P* < .01). - Had greater range of head and neck motion (*P* < .01)	At follow-up (n = 45): −13.3% of patients deteriorated −66.67% of patients same −20.0% of patients improved Of note, 10 deaths at study follow-up with 1 patient death attributed to CSM.
Bednarik et al^7^ Kadanka^8-11^ RCT	N = 33 Mean Age = 54 yr Male = 74%	2 yr (NR) 3 yr (90%) 10 yr (78%)	1. Clinical signs and symptoms of cervical cord Dysfunction 2. MRI criteria for cervical mono- and multisegmental cord compression and/or myelopathy due to spondylosis (including soft disc herniations) with or without developmentally narrow spinal canal 3. Age < 75 yr 4. mJOA score > 12 5. Patient’s consent to surgery	Change in mJOA score Subjective patients own evaluation 10m walk test Score of daily activities Electrophysiology	Deterioration or non-response to conservative treatment at 3-years (*P* < .05): -Younger age -Lower Torg-Pavlov ratio -higher mJOA -taller height -longer CMCT (abductor digiti quinti)	Follow-up at (based on mJOA): 1-year 15.1% deteriorated 2-year 34.5% deteriorated 3-year 26.7% deteriorated Subjective deterioration: 10-year: 56% vs 45.5%(surgical) Of note, 17 deaths at 10-years with no death attributed to CSM.
Lees and Turner^12^ Prospective cohort	N = 44 Mean age = NR (Range, 21-80 yr) Male = 68%	5 yr (100%)	1. Radiological and myelographic evidence of cervical spondylosis with signs of cord damage 2. Extensor plantar responses 3. All patients with other neurological diseases such as disseminated sclerosis, even if spondylosis was also present, were excluded	No scale used	Not assessed	14.3% (4/28) with collar treatment worsened and 3/4 were eventually operated (conversion to surgery 10.7%) Of note, 10 deaths during study period with 2 deaths attributed to CSM.
Matsumoto et al^13,14^ Retrospective cohort	N = 52, N = 27 Mean age = 55 yr Male = 75%	3 yr (NR) 4 yr (NR)	1. Diagnosed to have cervical compressive myelopathy based on both neurological examination and MRI findings showing spinal cord compression 2. Mild paresis 3. JOA ≥ 10	JOA MRI factors	No MRI factors predicted outcome	Follow-up at (based on JOA): 3-year 31% deteriorated 4-year 37% deteriorated
Nakamura et al^15^ Retrospective cohort	N = 64 Mean age = 52 yr (Range, 32-73) Male = 72%	6 yr (83%)	Motor function disability in the upper or lower extremity or in both (Based on the motor function evaluation of the JOA)	Motor JOA	Assessed, but no significant factors associated with worsening	Follow-up at 6 years −3% Deterioration of Lower limb motor function
Oshima et al^16^ Retrospective cohort	N = 45 Mean age = 59 yr (Range, 35-76) Male = 60%	6.5 yr (82%)	1. Motor function JOA scores of ≥ 3 in both upper and lower extremities 2. Cervical spinal cord compression with ISI on T2-weighted MRI	Motor JOA	More likely to undergo surgery - Local Slip OR 4.7 (1.67-13.0) - Segmental lordotic angle <0° OR 4.5 (1.59-12.8)	Follow-up at 6.5 years −40% Deterioration of motor function
Roberts^17^ Retrospective cohort	** N = 24 Mean age = 54.2 yr (Range, 41-69) Male = 75%	3 yr (86%)	1. Myelography diagnosis 2. Immobilization of the neck in a plastic or metal frame collar preceded by 2- to 3-wk bed rest in hospital	Motor disability: 1 = moderate inconvenience in normal daily activity 2 = activities severely limited but able to get about alone 3 = inability to get about without help 4 = bed- or chair-bound.	-No patient without improvement within 5 months of starting treatment improved with continued collar immobilization.	33% (n = 8) worsened, 37.5% unchanged, 29.2% (n = 7) improved based on motor disability grading.
Sampath et al^18^ Prospective cohort	N = 31* Mean age = 48.7 yr (Range, 21-75) Male = 48%	1 yr (74%)*	1. Consultation sought for treatment, not second opinion 2. ≥ 8 weeks of symptoms consistent with cervical spondylosis 3. Radiographical evidence of spondylosis 4. ≤ 1 prior surgical or intradiscal procedures 5. Able to read English at ≥ 8th grade level and fluent in spoken English 6. Age > 18 yr 7. Absence of ailment preventing participation 8. Legal US residence, no incarceration, signed informed consent	Number of symptoms Patient satisfaction Pain severity Activities of daily living	Not assessed	Follow up at 1-year: - Average worsening of activities of conservatively treated patients (*P* < .05)
Shimomura et al^19^ Sumi et al^20^ Prospective cohort	N = 70 ††, N = 60 ‡‡ Mean age = 55.1 +/− 11.8 yr Male = 70%	3 yr (80%) †† 6.5 yr (79%) ‡‡	Mild CSM (mJOA ≥ 13)	JOA MRI factors	Extent of cord compression predicted worsening OR 26.6 (1.7-421.5)	Follow-up at 3-year (JOA): −19.6% deterioration
Yoshimatsu et al^21^ Retrospective cohort	N = 69* Mean age = 67 yr (Range, 42-87) Male = 51%	2.5 yr (NR)	1. CSM based on clinical signs and the presence of compression on the spinal cord by MRI 2. Patients self-selected to be in the conservative treatment group after treatment opinions were explained to them 3. All patients except 2 had an initial JOA score ≥ 13	JOA	Increased duration of symptoms was related with clinical deterioration of symptoms (*P* = .001) Patient who did not follow rigorous conservative treatment were more likely to deteriorate (*P* < .025)	Follow-up at 2.5-year (JOA): −62% deterioration
Wu et al^22^ Retrospective cohort	N = 14 140; mean age = NR; % male NR	>1 year; % NR	Subjects hospitalized and discharged with the diagnostic ICD-9 code for CSM (721.1) (National Health Research Institute of Taiwan)	Incidence of Spinal Cord Injury	Not assessed	- Incidence Risk for hospitalization for SCI 13.9/1000 person-years (11.6-16.6) for patients with CSM
Wu et al^23^ Retrospective cohort	N = 5604; mean age = 60.35 + 14 years; 70% male	>3 years; % NR	Subjects hospitalized within the study period with a first-time discharge summary containing the diagnostic ICD-9 code for OPLL (723.7x) (National Health Research Institute of Taiwan) Patients hospitalized for OPLL who have not received spinal intervention within the previous 6 months (National Health Research Institute of Taiwan)	Incidence of Spinal Cord Injury	Not assessed	- Incidence Risk for hospitalization for SCI of 4.8/1000 person-years with OPLL. - Rate of hospitalization for SCI in patients with DCM from OPLL was higher than the rate observed in a healthy population (0.18/1000 person-years; hazard ratio = 32.2; 10.4-99.0; *P* < .001).
Matsunaga et al^24^ Prospective cohort	N = 36; mean age = 61.8 years; 59% male	17.6 years (Range = 10-30 years); % NR	Patients with DCM from OPLL	JOA Nurick	Not assessed	- Increased myelopathy was observed in 64% (23/36) patients
Martin et al^25^ Ambispective Cohort	N = 117; mean age 54.6 years; 54% male	2.6 years (100%)	Patients seen in surgical consultation with DCM with: 1) newly diagnosed (N = 95) or 2) recurrent myelopathy (N = 22) after previous surgery	Surgeon’s assessment mJOA QuickDASH JAMAR grip GRASSP-Myelopathy Electronic gait analysis Berg balance Anatomical MRI	mJOA severity category	- Neurological deterioration observed in 57% patients with primary DCM; 73% with recurrent DCM.

### Natural History of the Cervical Degenerative Process

**Figure 1.**
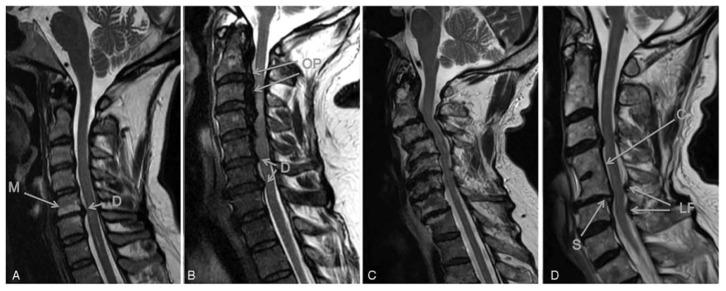
Spectrum of changes in DCM represented by T2 anatomical MRIs. A, A single-level disc degeneration resulting in spinal cord compression (D). Also shown here are hyperintensity changes of the vertebral body endplates consistent with type I or II modic changes (M). B, A patient with ossification of the posterior longitudinal ligament (OP) and disc degeneration (D). C, A patient with severe multi-level bone and disc degeneration and kyphotic deformity. D, A patient with congenital fusion between C4-5 (C). In addition, there is a retrolisthesis evident at the inferior end of the fused vertebrae (S) as well as enlargement of the ligamentum flavum (LF). Taken from Nouri et al.^34^

As with all osteoarthritic disease, cervical spine related degeneration is principally a function of use-intensity, genetics, environmental/lifestyle factors and time.^1,26^ This process begins at the intervertebral disc (IVD), wherein decreased compliance occurs secondary to a reduction of nucleus pulposus hydration and fibrous transformation. The early phase of this transformation, highlighted by intranuclear cleft formation, a precursor to future more extensive degenerative disease, is seen frequently in young asymptomatic individuals as early as the third decade.^26-30^ In addition to fibrous transformation, the height of the IVD progressively decreases, often in asymmetric fashion, leading to an unequal distribution of forces across the endplates, ultimately resulting in remodeling of the vertebral bones.^31^ This remodeling takes the form of increased antero-posterior length and decreased vertebral height, osteophyte/bone spur formation, and in certain circumstances can lead to disc herniation into the vertebrae (Schmorl’s node) and adjacent vertebral autofusion.^27^ These disc related degenerative changes can have a number of downstream consequences contributing to eventual myelopathy: 1) ligamentous changes including in-folding of the ligamentum flavum, reactional hypertrophy, calcification, and ossification of the posterior longitudinal ligament and ligamentum flavum; 2) cervical alignment changes, including development of kyphosis, scoliosis, hyperlordosis or listhesis, and; 3) reduction in cervical canal size, with progressive decrease in the space available for the spinal cord,^27,31,32^ Figure 1. In addition, it has also been recently shown that aberrations in the paraspinal muscle morphology, including fatty infiltration, presents as part of the degenerative process.^33^

While most individuals will develop some degree of degenerative change within the cervical spine with increased age, most experience few symptoms. However, in a limited proportion of the general population, severe forms of the aforementioned changes can result in spinal cord compression and myelopathy development. Another group may experience significant degenerative changes at a localized level, such as a single disc, with otherwise global preservation of cervical anatomy.

### Risk Factors for Cervical Spine Degeneration and DCM Development

Given that the development of degenerative disc disease is an age-dependent process, the principle risk factor for DCM development is age, with an average age of onset in the largest cohorts is typically approximating the mid 50 s to 60 s years of age.^35-37^ In addition, males seem to be relatively overrepresented in many of the largest DCM cohorts (representing approximately 2/3rds of patients in the global AOSpine study of operated DCM patients),^36^ suggesting that males may be at an elevated risk for DCM development. This is supported by a large Taiwanese study showing that the highest incidence of DCM for both males and females occurred in their 70 s, but with a significant difference in incidence rates between the genders (28.9 for males vs 15.3 for females per 100 000 person-years).^22^ Some research has suggested this may be due to anatomical variations in canal/vertebral-body ratio, but studies on this subject are sparse.^38^ Clinical series of surgically treated DCM patients have shown that males more commonly present with more severe degenerative states, more commonly have multilevel compression, and T2 hyperintensity changes.^34^ The nature of this association, however, is not completely clear and may be explained by multiple factors including exposure to certain work-related or environmental factors in males as compared to females.

Several other risk factors for cervical spine degeneration and DCM development have been investigated in the literature, with definitive evidence for causal factors remaining limited.^1,39^ The most relevant of these factors are discussed in further detail below.

#### Anatomical cervical cord-canal mismatch

Intuitively, the congenital presence of a narrow spinal canal, also known as “congenital stenosis” or “developmental canal stenosis” should predispose individuals to the development of DCM. However, evidence supporting a clear association between congenital stenosis and myelopathy development remains sparse.^39^ Older criteria for defining a narrow canal anatomically based on radiographs and cadaver studies set a sagittal width of <12-13mm or a Torg-Pavlov ratio <0.80-0.82 for the diagnosis.^40-43^

**Figure 2.**
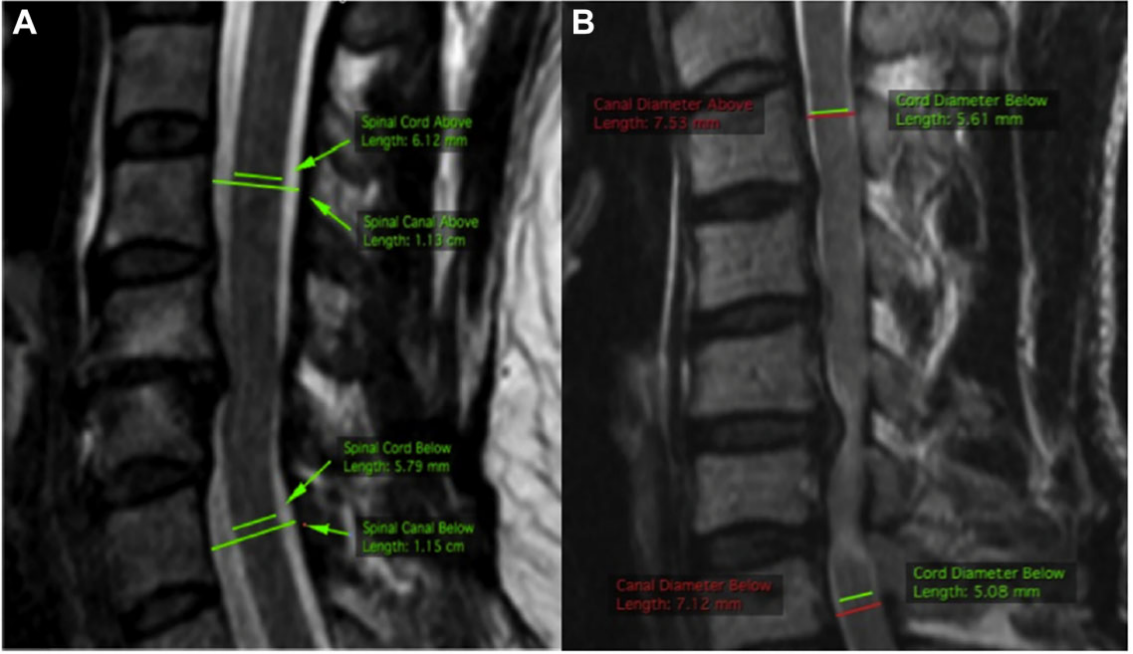
Cord-Canal mismatch measurement in 2 different patients based on sagittal T2 MRI. A, Represents a patient without a cord-canal mismatch with a SCOR calculated at 52.2% ([6.12 + 5.79]/[11.3 + 11.5]) × 100. B, The patient has a cord-canal mismatch as evidenced by an SCOR of 73.0% ([5.61 + 5.08]/[7.53 + 7.12]) × 100. Taken from Nouri et al.^44^

While previous research has focused primarily on canal size, recent studies have recognized that spinal cord size also varies and have thus argued that relative size of the canal and cord should be assessed.^44,45^ The basis of a cord-canal size mismatch is that both a narrow canal and a large spinal cord can predispose patients to cervical spinal cord compression and potential myelopathy development.^44,46^ This knowledge has resulted in the development of relative parameters based on MRI that incorporate the size of the spinal cord, including: space available for the cord (SAC) and spinal cord occupation ratio (SCOR), Figure 2. Depending on the technique, a cord-canal mismatch can be defined as a SCOR ≥70% when measured on the midsagittal plane,^47^ ≥80% on the axial plane,^48^ or <5mm of SAC.^49^ While it has been shown that both the large cord and smaller canal are risk factors for DCM, it has likewise been shown that there is greater anatomical variability in canal size compared to spinal cord size in the population, indicating that this will be the more common reason for a cord-canal mismatch.^44^

The risk of spinal cord compression in patients with a cord-canal mismatch has been attributed to 1) less space within the canal, which lowers the amount of degenerative changes or migration of spine structures into the canal that are necessary for spinal cord compression to occur, 2) less cerebrospinal fluid cushion that surrounds the spinal cord, which decreases the ability of the fluid to absorb kinetic forces directed at the spine throughout movement of the head and neck.^44^

In the sub-analysis of the international and multicenter AOSpine studies on patients with DCM surgically treated, the prevalence of a cord-canal mismatch using a sagittal SCOR ≥70% was found to be 8.4%, and patients diagnosed with a cord-canal mismatch at non-compressed sites were found to be 5.4 years younger and presented reduced baseline neurological function and quality of life.^47^

Future research is needed to understand the role of genetic causes of cord-canal mismatch and to gain increased insight into how mismatch influences myelopathy development and/or progression.

#### Congenital cervical fusion (Klippel-Feil syndrome)

**Figure 3.**
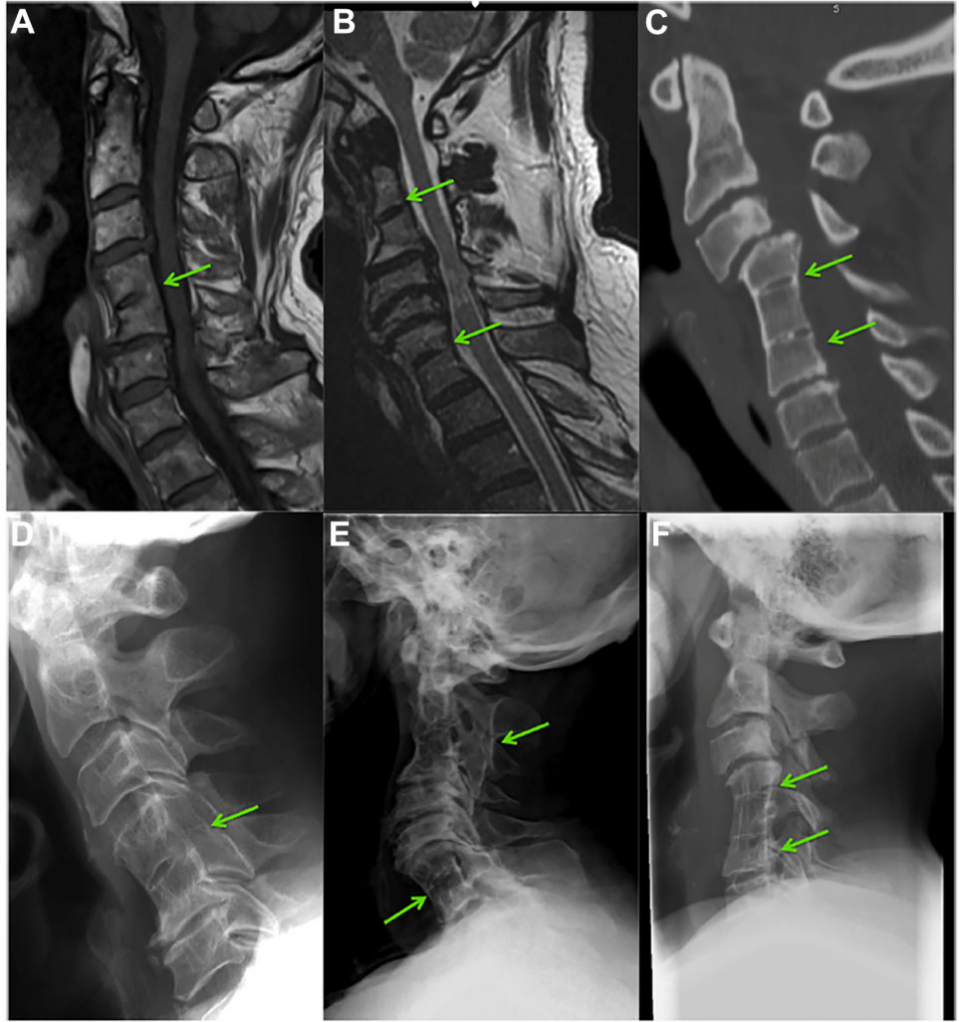
Klippel-Feil syndrome and degenerative cervical myelopathy. A and D, A single fusion of C4-5 seen on T1 MRI and lateral radiograph of the same patient. B and E, Two non-contiguous fusions between C2-3 and C6-7 on T2 MRI and lateral radiograph. C and F, Two contiguous fusions between C4-5 and C5-6 on CT and lateral radiograph. Adapted from Nouri et al.^53^

Congenital fusion of cervical vertebrae, which can be seen in the context of Klippel-Feil Syndrome (KFS) Figure 3, has a reported prevalence of between 0.5 and 0.7% based on cadaver and imaging studies.^50,51^ Although KFS is classically associated with the triad of a short neck, lower posterior hairline, and restriction of neck movement, all three of these features are only present in a minority of cases.^51,52^ Most commonly, congenital cervical fusion is encountered incidentally without any of the other classical clinical features of KFS.

It has been previously hypothesized that patients with congenital fusions are at an increased risk for myelopathy development at the segments adjacent to fusion^53-55^ because fusion may increase the biomechanical stress on the adjacent discs and accelerate degeneration.^56,57^ A small study has shown a relatively high prevalence rate of congenital fusions among DCM patients (2.4%) compared to the general population.^53^ However, no definitive studies exist to establish a clear link between congenital cervical fusions and increased predisposition to DCM. The same study also showed that in patients with congenital fusions, adjacent segment disease was preferentially present at segments toward the center of the cervical spine; however, despite the higher prevalence rates among DCM patients, and considering the limited size of the patient population with congenital fusion, no difference in duration of symptoms or age was found.^53^

#### Genetic factors

Alterations in gene structure and expression are known to contribute to disease. Several studies have investigated for genetic factors associated with DCM, with the current evidence supporting a genetic basis for development of this condition. The most convincing study of an underlying genetic predisposition was undertaken by Patel et al^58^ who utilized population-based data and cross referenced a genealogic database of over 2 million Utah residents with 10 years of clinical diagnosis data from a large tertiary hospital. They showed, using the Genealogical Index of Familiality, a significant excess relatedness for disease with the relative risk for DCM among first-degree relatives to be 5.21. While this study did not identify specific genes of interest, it demonstrated that heritability plays a role in DCM development.

Systematic reviews on genetic factors have supported the principle of a genetic predisposition to both DCM development and clinical severity.^59,60^ Some genetic polymorphisms have been linked to disc degeneration and spondylosis, while others are linked to OPLL development. The most recent systematic review and meta-analysis has identified 28 genes of interest with regards to DCM, including those affecting collagens,^27,30,32^ Interlukins,^1,37,34^ Transforming growth factor,^1,2,3^ Vitamin D binding protein, Bone morphogenic protein,^2,4,30^ Fibroblast growth factor (1, 2) as well as many others.^60^ From these, 22 genes were found to be associated with radiologically defined spinal pathology, predominantly OPLL, 12 associated with clinical DCM development, 8 were found to have an effect on the radiological severity, 3 had an effect on clinical severity, and 6 on the clinical response to surgery in spinal cord disease.^60^ However, the specific mechanisms by which these genetic factors affect the natural history remain incompletely defined because none of the candidates have been studied sufficiently to provide a high level of evidence, and most studies have been conducted in isolated populations (almost all of the studies have been conducted in China, Japan, and South Korea).^60^ While these genetic studies have predominately focuses on polymorphisms of specific genes, recent research has shown that expression of specific microRNA’s can be applied clinically as a biomarker in the clinical setting.^61^ Laliberte et al^61^ has recently shown that greater mir-21-5p expression was associated with worse surgical prognosis based on the mJOA at 1 year follow-up. The authors attribute this effect of mir-21-5p on its presumed pro-inflammatory mechanism.

While the genetic basis for certain syndromic conditions associated with structural aberrations of the cervical spine is better described, understanding surrounding relative susceptibility to myelopathy development with most of these conditions remains incomplete. As examples, patients with Klippel-Feil Syndrome, Down’s Syndrome (atlanto-axial abnormalities),^62^ Ehlers Danlos Syndrome (Hypermobility)^63,64^ and Achondroplasia (congenital cervical stenosis)^65^ have an inherited predisposition for cervical spine anomalies, many of which can lead to cervical canal narrowing. However, the relative risk of symptomatic myelopathy development among patients with these conditions, as compared to nonaffected individuals, remains largely unknown.

Given the current evidence, genetic factors likely influence DCM development, severity, and recovery potential, however, validation studies with genetically distinct populations will need to be undertaken before these research findings can be applied to clinical practice.

#### Role of minor trauma, cervical instability and motion on the development of DCM and spinal cord injury

Although clinical deterioration may occur spontaneously, DCM patients are at risk of developing acute spinal cord injury (SCI) in case of physical trauma (i.e. fall or motor vehicle accident). This increased risk is thought to be secondary to several factors, including the presence of spasticity and gait unsteadiness that increase the propensity for falls, and the presence of pre-existing canal narrowing and spinal cord compression. A recent systematic review showed that the incidence of hospitalization for SCI in Taiwan was 13.9 and 4.8 per 1000 person-years in patients with DCM and myelopathy secondary to OPLL, respectively.^22,5,23^ The rate of hospitalization of SCI in patients with myelopathy from OPLL was significantly higher than the rate observed in a healthy population (hazard ratio of 32.2).^5^ Contrary to these findings, a prospective study by Bednarik, Sladkova^66^ did not show a relationship between traumatic events and myelopathy onset in 199 patients with initial asymptomatic spinal cord compression. During the study, 14 patients experienced traumatic events at an average of 44-months follow-up, but none were associated with immediate neurological deterioration.

**Figure 4.**
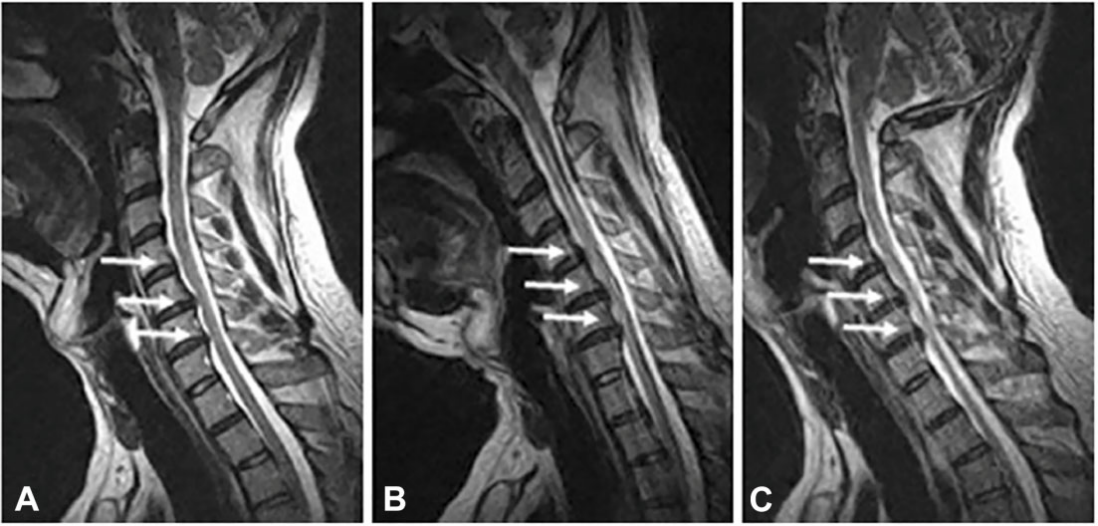
Dynamic cervical spinal cord compression on MRI. A neutral (A) and flexion (B) and extension (C) T2 MRI showing the effect of movement on spinal cord compression. Here flexion of the spine unmasks spinal cord compression not clear in neutral imaging. Taken from—Lao et al.^71^

Aside from more dramatic or catastrophic instances of traumatic SCI, it has been suggested that minor traumatic events, leading to significant head and neck movements, may cause episodes of decline or may be the precipitating event causing neurological deterioration in patients with known DCM.^1,66-68^ Movement-based spinal cord trauma can be explained by changes in the cross-sectional diameter of the spinal canal during flexion and extension,^69^ Figure 4. In a cohort of patients with neck pain, with or without neurological symptoms, studied with dynamic MRI, Hayashi, Wang^70^ reported a high level of missed stenosis in the neutral position, and that dynamic stenosis was discovered in 8.3% of vertebral segments only when in extension, and 1.6% only when in flexion. They noted that missed stenosis occurred most commonly at the C5-6 segment. Cervical range of motion in those with stenosis has also been implicated as a factor. Matsunaga, Kukita^68^ showed that myelopathy was present in all patients with OPLL with stenosis <6mm and that no myelopathy was present in those with canal diameters of ≥ 14mm; however, when the canal diameter was >6mm but <14mm, myelopathy preferentially developed in those with increased range of cervical motion.

Repetitive spinal cord compression events resulting from cervical instability in the setting of cervical spondylolisthesis has also been suggested to be a potential marker of worse disease severity and as a potential cause of neurological deterioration in DCM patients. This was recently highlighted by a sub-analysis of the AO Spine International studies on DCM, showing that patients with spondylolisthesis present with worse neurological function at baseline, and when propensity matched on other key variables, presented with worse neurological outcomes than patients without spondylolisthesis.^72^ A recent systematic review on this topic corroborated these results.^73^ It is possible that this subset of patients undergoes an alternative natural disease course highlighted by an accumulation of minor traumatic events; this, however, remains speculative.

### Natural History of Non-Myelopathic Spinal Cord Compression (NMSCC)

As discussed above, age-related cervical degenerative changes occur commonly, often leading to some degree of spinal canal narrowing or spinal cord compression. However, most patients with spinal canal narrowing or spinal cord compression do not have clinical signs and symptoms of myelopathy [non-myelopathy spinal cord compression (NMSCC)].

**Figure 5.**
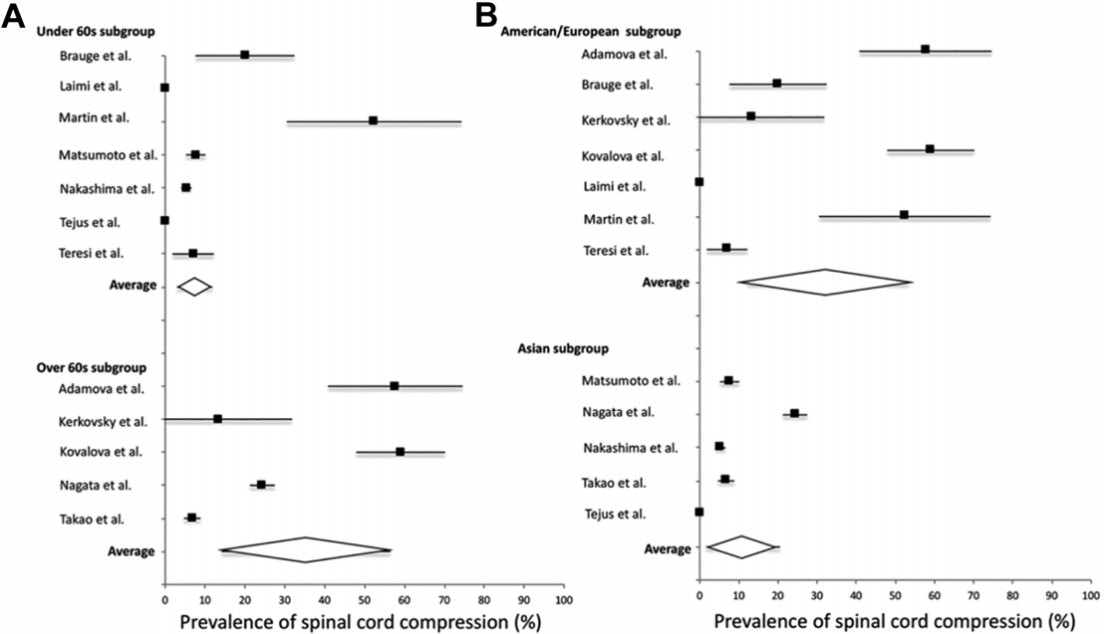
Prevalence of asymptomatic spinal cord compression among different demographic groups. Taken from Smith et al.^76^

In a Japanese MRI study of 1,211 asymptomatic volunteers ranging between the 2nd and 7th decade, NMSCC was observed in 5.3% of the study participants, with a second Japanese study finding cord compression in 7.6% of 497 asymptomatic persons undergoing MRI.^26,45^ However, more recent studies have shown a much higher incidence of asymptomatic spinal cord compression, particularly in older patients. Kovalova, Kerkovsky^74^ noted NMSCC in 57.9% of 183 volunteers older than 40 years undergoing cervical MRI. Similarly, a sub-analysis of 40 non-myelopathic control subjects in a prospective DCM imaging study discovered that 20 of these asymptomatic patients had MRI evidence of spinal cord compression (defined as indentation, flattening, or torsion).^75^ Furthermore, the latter study showed that these NMSCC patients had macrostructural and microstructural changes (based on advanced imaging techniques) similar to those observed in symptomatic DCM. The large discrepancy in prevalence rates between these studies is challenging to interpret. However, it is likely that variations in diagnostic criteria for NMSCC between studies, as well as differences between the ethnic populations studied, may explain the heterogeneity. Specific prevalence rates of asymptomatic spinal cord compression among different study population demographics have recently been presented in a meta-analysis,^76^ Figure 5. Based on this analysis, the prevalence of NMSCC in a healthy population is 24.2%, and 35.3% in individuals >60 years.^76^

The key question when considering patients with NMSCC relates to their likelihood of developing myelopathy over time. Of the 20 NMSCC patients discussed in the imaging study above, 2 (10%) eventually developed symptoms of myelopathy at a median follow-up of 21-months. In the largest prospective study performed to date on this topic, Bednarik et al found that among 199 patients enrolled with NMSCC, 8% at 1-year follow-up and 22.6% at a median of 44 months follow-up (Range 2-12 years) developed symptoms of myelopathy.^77^ In this study, factors shown to be predictive of myelopathy development in multivariate survival analysis at 1-year follow-up included: the presence of clinical cervical radiculopathy, prolonged somatosensory and motor evoked potentials, and the absence of spinal cord T2 hyperintensity on MRI. Interestingly, at longer-term follow-up (44-months), the presence of T2 hyperintensity on MRI portended a higher risk of myelopathy development. Hence, the importance of T2 signal change in predicting the risk of clinical progression remains unknown. In another study by the same group in 2017, wherein 13.4% of patients (15/112) developed DCM at a median follow-up of 36-months, multivariate analysis showed that radiculopathy, axial cross-sectional area ≤ 70.1mm2, and compression ratio (CR, anteroposterior size/transverse size of the cord on axial imaging) ≤ 0.4 ≤ 0.4 was predictive of DCM development.^78^ The difference in the rate of progression and significant predictors could be influenced by a different recruitment strategy of NMDCC patients in these studies. The former study included patients referred to a center for radiculopathy or cervical pain (i.e. without myelopathic symptoms or signs, but not clearly asymptomatic) and has more severe radiological compression in contrast to the latter one recruited randomly as a part of the epidemiological study. While it is notable that many of the predictors reported have differed with follow-up time, the presence of radiculopathy has remained a consistent predictor across studies. In addition to clinical radiculopathy, electrophysiological measures, including prolonged SSEPs and MEPs, are associated with an increased rate of myelopathy development, and their presence has been suggested as a potential indication to consider surgery for patients with NMSCC.^79^

Further work is needed to understand the prevalence of NMSCC more precisely, as well as rates of deterioration, and to identify key biomarkers (i.e., clinical, imaging, genetic, and electrophysiological factors) that predict clinical course for purposes of aiding clinical communication, facilitating treatment decisions, and gauging the optimal follow-up interval for those who are observed over time. Ultimately, this information would be critical to direct updates to the guidelines on the management of these patients, which are currently based on limited evidence.^80^

### Progression of Asymptomatic OPLL and Myelopathy Development

While the prevalence of OPLL varies significantly depending on the region of the world and ethnicity considered (approximately 1.3% among Caucasians and 6.3% among Japanese), clinical experience dictates that only a fraction of patients with this ligamentous aberration are symptomatic and require treatment.^81^ From a radiographic progression perspective, a recent retrospective cohort study from Japan reported on 109 individuals with incidentally discovered OPLL. At a mean follow-up of 80.8-months, the incidence of OPLL progression was 18.3%, defined as an increase of >2mm in the sagittal thickness and/or the length of the ossification.^82^ Risk factors for progression included younger age at diagnosis, higher serum uric acid levels, OPLL involvement of ≥ 3 vertebral levels, and continuous type of OPLL, whereas progression was less common in individuals with a segmental type of OPLL.^82^ Another study of conservatively treated OPLL patients with no or “slight” myelopathy also found that younger age was a significant predictor of OPLL progression, in addition to higher body weight and BMI.^83^

In another recent study, Park et al^84^ reported a progression rate of 26.8% in vertical dimensions and 22.7% in antero-posterior dimensions after a mean follow-up of 39.3 months. In this study, younger age at diagnosis and presence of OPLL at C2-3 were found to be among the variables predicting higher risk of radiographic progression. They also noted that segmental progression of an ossified mass occurred with increased segmental range of motion (≥5°).

Although radiographic progression of OPLL is of importance, the greater clinical concern relates to the risk of myelopathy development among patients with asymptomatic OPLL. Unfortunately, estimates surrounding this question are extremely imprecise and based on low-quality evidence. A previous systematic review^79^ and recent studies have reported a large range of myelopathy development, ranging from 0% in subjects (0/27) followed for a mean of 59 months^85^ to 61.5% in subjects (96/156) with a mean 123.6-month follow-up.^86^ In addition, the recent study by Park et al^84^ demonstrated that 9.3% of patients had mild myelopathy at a mean follow-up of 39.3 months and that an additional 2.1% of patients were operated on myelopathy during the study period. Risk factors for myelopathy development included canal stenosis of ≥60%, lateral deviated OPLL, and increased cervical range of motion.^86^

### Progression of DCM in Non-Operatively Treated Patients

When considering the topic of DCM, perhaps the greatest clinical knowledge gap relates to the management of patients with mild DCM. Recent guidelines suggest either surgery or clinical observation to be reasonable initial treatment options.^80^ The central question underlying our treatment decision for mild DCM patients is: what will happen if we do not intervene with surgical decompression? Apart from mild patients, more severely affected patients, for a variety of reasons, may also not undergo surgery; for purposes of quantifying expectations for the future, attaining a sound understanding of natural history for this group is important.

The sparse and largely low-quality evidence currently available provides imprecise estimates surrounding the expected clinical course for patients with symptomatic myelopathy treated non-operatively. Systematic reviews of the literature have shown that conversion to surgery for non-operatively followed patients ranged between 4% to 40% over 3-7 year follow-up periods.^5^ From the perspective of clinical progression, the available literature suggests that 20-62% of DCM patients treated non-operatively will experience neurological deterioration as assessed by the mJOA over 3-6 years of follow-up.^87^ However, most of these studies used JOA or mJOA to define neurological progression without considering the minimal detectible difference (MDD), which appears to be greater than 1 point based on reliability studies.^88,89^ A recent study using an array of measures of spinal cord function found that 57% of DCM patients deteriorate over a mean follow-up of 2.5 years, with quantitative measurement of hand grip strength, hand dexterity, electronic gait analysis, and balance showing the greatest sensitivity to deterioration.^25^ Factors influencing the imprecision of current estimates include significant between study variations in duration and rates of follow-up, definition of clinical change or deterioration, and the specifics of cohort composition as it relates to the severity of symptoms and underlying pathology. The inexact nature of these estimates leaves clinicians in a difficult situation when attempting to counsel patients about the relative merits of operative vs. non-operative treatment.

In addition to understanding rates of clinical progression, it is also important for clinicians to understand predictors of deterioration with non-operative care so that those at the highest risk may be selectively targeted with early surgery. While several studies have investigated potential predictors of neurological deterioration, few variables have reliably demonstrated importance in this regard.^16,19,90^ Circumferential compression of the spinal cord has been shown to be predictive of myelopathy progression.^90,19^ Likewise, an increased range of motion, which interestingly has also been related with OPLL progression, as previously noted, has also been suggested to predict neurological decline.^16^ From an electrophysiological perspective, normal central motor conduction time has been shown to predict lack of neurological decline in mild myelopathy patients treated without surgery.^90^ Other factors, such as age and the presence of T2 hyperintensity on MRI have not reliably predicted the clinical course of patients treated non-operatively.^87,90,19^

In a recent study, quantitative MRI (qMRI) techniques, including white to gray matter ratio, fractional anisotropy and cross-sectional assessment were shown to detect myelopathy progression (Progression was defined as patients’ subjective impression, 2-point mJOA decrease, ≥3 clinical measures worsening ≥5%, increased compression on MRI, or ≥1 of 10 qMRI measures or composite score worsening) with a higher sensitivity than mJOA.^91^ This study highlighted that while patients may seem stable neurologically by conventional measures, disease progression not appreciated by less sensitive clinical measures may be occurring.

Although a significant proportion of patients with DCM treated non-operatively will deteriorate over time, it is also clear within the literature that a sizable proportion will remain stable over time. A new line of evidence is emerging that may help to explain this clinical stability, showing that “supra-spinal” and cortical changes may facilitate adaptation of neurological function.^92,93^ It has been recently suggested that a “functional reserve capacity,” which is facilitated by new cortical motor connection in the supplementary motor region, may provide a compensatory mechanism in patients with spinal cord compression and may mask spinal cord sufferance.^92^ While further work to support these findings is necessary, such a mechanism may help to explain the clinical stability, or even occasional clinical improvement, in neurological status seen in DCM patients, despite ongoing spinal cord compression.

## Conclusion and Future Directions

Degeneration of the cervical spine progressing to spinal cord compression, and subsequently development of myelopathy, represents a continuum of disease progression that remains incompletely understood. Furthermore, despite the ubiquity of spinal cord compression due to degenerative cervical spine disease, our understanding surrounding the frequency of clinical deterioration with non-operative care—the natural history of this condition—remains limited. This knowledge gap hinders clinicians’ ability to adequately counsel patients. A number of ongoing studies are underway to address this knowledge gap including a Canadian multicenter prospective longitudinal study that assesses the natural history of patients with mild myelopathy treated with initial non-operative care (DCM-NH study). Started in 2019, and now at about 30% of the 220 subject target sample size, this study will follow mild DCM patients for up to 5 years to understand the true rate of clinical change for this patient group. This study, in addition to others, also investigates the utility of microstructural MRI variables, in addition to blood biomarkers, to predict the clinical trajectory of DCM patients treated non-operatively. In addition to clinical, imaging and blood biomarker related variables, electrophysiological parameters such as sensory and motor evoked potentials are becoming increasingly used and reported as a means to assess spinal cord sufferance and predict clinical course.^2,3^ It is anticipated that incorporation of these and other tools may permit a more individualized estimate for disease progression facilitating personalized treatment recommendations for DCM patients based on their specific risk for clinical deterioration.
